# Comparing outcomes of balloon-expandable vs. self-expandable valves in transcatheter aortic valve replacement: a systematic review and meta-analysis

**DOI:** 10.1097/MS9.0000000000001743

**Published:** 2024-01-26

**Authors:** Qaisar Ali Khan, Ameer Mustafa Farrukh, Naod F. Belay, David Li, Muhammad Afzal, Adithya Nadella, Bader Semakieh, Abdul Baqi, Alondra M. Robles Rodríguez, Meryem Biougnach

**Affiliations:** a Khyber Teaching Hospital, MTI KTH, Peshawar, Pakistan; b University of Galway School of Medicine, Galway, Ireland; c Michigan State University, East Lansing, MI; d Idaho College of Osteopathic Medicine, Meridian, ID; e St. George’s University School of Medicine, True Blue, Grenada; f Nanjing Medical University, Nanjing, China; g Arkansas College of Osteopathic Medicine, AR; h Mercy Saint Vincent Medical Center, Toledo, OH; i Universidad Central del Caribe, PR; j Lycee Paul Valery High School, Meknes, Morocco

**Keywords:** Aortic stenosis, aortic valve, aortic valve replacement, ballon-expandable valves, self-expanding valves, transcatheter valve replacement

## Abstract

**Background:**

Aortic stenosis (AS) is a common heart condition categorized into congenital and acquired forms. Transcatheter aortic valve replacement (TAVR) is an innovative method for AS management, and two valve types, self-expanding valves (SEV) and balloon-expandable valves (BEV), are used in TAVR. The objective of this study is to compare the clinical outcomes associated with balloon-expandable and self-expandable valves in transcatheter aortic valve replacement.

**Methods:**

The researchers conducted a comprehensive meta-analysis following PRISMA guidelines and AMSTAR-2 tool. The methodology involved a systematic literature search, strict eligibility criteria, unbiased study selection, meticulous data extraction, quality assessment, and rigorous statistical analysis.

**Results:**

Our analysis included twenty-six papers and 26 553 patients. BEV exhibited significant advantages over SEV in overall mortality across 21 studies, particularly in perioperative and 30-day assessments. However, no substantial disparities emerged between the two valve types in stroke incidence. BEV demonstrated notable benefits in reducing hospitalization rates across 6 studies and significantly fewer instances of permanent pacemaker implantations across 19 studies, particularly evident in the perioperative setting. Other secondary outcomes like bleeding, acute kidney injury, and myocardial infarction showcased non-significant differences between BEV and SEV.

**Conclusion:**

The analysis indicates that BEV may offer benefits in specific aspects of TAVR outcomes, but further research is needed to fully understand the factors influencing patient outcomes and mortality in TAVR procedures.

## Introduction

Highlights

- In this systematic review, we have compared the outcomes of balloon-expandable vs. self-expandable valves in transcatheter aortic valve replacement.
- Our findings suggest potential advantages of balloon-expandable valves in terms of mortality and fewer hospitalizations.
- Further research is still warranted to evaluate the two options, particularly considering the non-significant results for cost-effectiveness and patient-reported outcomes.

Regarded as the most common cause of left ventricular outflow obstruction, aortic stenosis (AS) affects at least 5% of the population, with incidence rates increasing with age^1^. From an aetiological perspective, AS is categorized into two types—congenital AS, where an individual has the disorder since birth, and acquired AS. In developing nations, rheumatic fever remains the leading cause of acquired aortic stenosis—the clinical manifestations of the disease range from dyspnoea on exertion to chest pain and fatigue. However, in some severe cases, AS can cause heart failure^2^.

Echocardiography remains the imaging modality for diagnosing aortic stenosis, with aortic valve replacement (AVR) being the treatment choice and is considered superior to medical management. Transcatheter aortic valve replacement (TAVR) has emerged as an innovative transformation in AVR surgeries, offering a less invasive alternative to traditional surgical valve replacement^3^. Over the last few decades, TAVR has witnessed a steep rise, revolutionizing the landscape of cardiovascular interventions. The fact that TAVR can deliver remarkable patient benefits, including reduced blood loss, fewer complications, and shorter hospital stays, among others, contributes to this. This minimally invasive procedure involves the insertion of a collapsible valve via a catheter, usually through the femoral artery or alternative access routes, negating the need for open-heart surgery. The valve is positioned within the native aortic valve annulus and then expanded or self-expanded (depending on the type) to replace the dysfunctional valve, thus restoring proper blood flow and alleviating symptoms.

While there are currently multiple options in the TAVR market, only two are approved by the Food and Drug Administration (FDA). They are CORE valves (Medtronic Fridley) and SAPIEN valves (Edwards Lifesciences)^3^. SAPIEN valves consist of bovine pericardial tissue and chromium cobalt alloy. SAPIEN valves can be balloon expanded. The EVOLUT-R is the newest generation Medtronic valve. It’s made of pig tissue with a nitinol frame. This valve is self-expandable (it does not require balloon inflation) and repositionable after deployment.

While multiple studies have provided valuable insights, they have often overlooked the broader picture. This comprehensive meta-analysis presents another perspective by combining and subgrouping outcomes within specific time frames. Through this study, we aim to contribute to the knowledge surrounding TAVR, offering a comprehensive assessment of the comparative advantages of self-expanding valves (SEV) and balloon-expandable valves (BEV). In doing so, we provide clinicians and researchers with valuable insights to inform their decision-making processes and optimize patient care in the dynamic field of transcatheter aortic valve replacement.

## Materials and methods

The researchers conducted this meta-analysis following the Preferred Reporting Items for Systematic Reviews (PRISMA) guidelines^4^. The work has been reported in line with the AMSTAR (Assessing the methodological quality of systematic review) tool.

### Literature search

Relevant articles were identified through Medline (via PubMed), Cochrane Library, Web of Science, and clinicaltrials.gov from inception to August 2023. Bibliographies of these studies were used to identify other relevant studies. The following search terms were used:

“Balloon-expandable,” “Transcatheter Aortic Valve Replacement,” “Self-expanding,” “Aortic Stenosis,” “Bioprosthesis,” “Valve in Valve,” “dysfunctional bioprosthesis,” “Aortic Valve replacement”.

### Eligibility criteria

The eligibility criteria followed the PICOS strategy:

Population: Patients undergoing TAVR via SEV or BEV.

Intervention: BEV.

Comparators: SEV.

Study design: Randomized controlled trials (RCT) and observational studies comparing the techniques.

Outcome: Studies that reported our outcomes of interest were included. These outcomes were:

Primary outcomes: Mortality, Stroke and Hospitalization

Secondary outcomes: Permanent pacemaker, bleeding, acute kidney injury, and myocardial infarction.

### Study selection and data extraction

All studies were screened for inclusion using the eligibility criteria detailed above. The selected articles were subsequently analyzed in their entirety by two of the investigators. This data were extracted into a pre-structured Microsoft Excel data sheet (Version 2019, Microsoft). Disagreements were resolved by consulting a separate author. The following data were extracted from the studies: First Author, the year of publication, country of origin, study design, age, gender, BMI (kg/m^2^), and outcomes of interest.

### Quality assessment

The relevant studies were assessed for quality via the Cochrane risk-of-bias (RoB2) tool^5^ for RCTs and the Newcastle–Ottawa Scale (NOS) for observational studies^6^. Two independent reviewers performed the quality assessment, and any discrepancy was resolved by consultation with another author.

### Statistical analysis

Data analysis was performed using the comprehensive meta-analysis (CMA) version 3.0. We presented dichotomous data as odds ratios (ORs) and continuous data as mean differences (MDs). A random-effects model was used to deal with the heterogeneity of included studies. An I2 index greater than 75 is demonstrated as high heterogeneity. A *p* value of less than 0.05 was considered statistically significant in all analyses.

## Results

### Literature search

**Figure 1:**
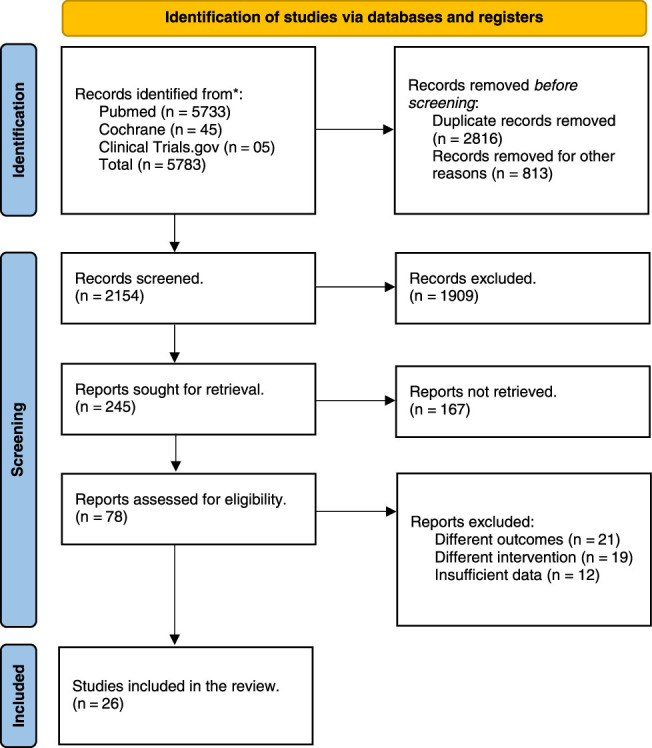
Preferred Reporting Items for Systematic Reviews and Meta-Analyses (PRISMA) flow chart of literature search.

A total of 5783 articles were retrieved. After removing duplicates, 2967 articles were screened via titles and abstracts. Ultimately, 78 articles were selected for in-depth review. Finally, the final qualitative and quantitative meta-analysis included 7 RCTs^7–13^ and 19 observational studies^14–32^. This selection process is illustrated in the PRISMA flowchart (Fig. 1). Moreover, the AMSTAR-2 checklist has been provided separately.

### Study characteristics

The characteristics of the included studies are shown in Table 1. There were 26 553 patients in the BEV group and 14 953 in the SEV group; hence, a total of 41 506 patients are included in this meta-analysis.

**Table 1:** Baseline characteristics of included participants

			BEV			SEV				
Author, year	Country	Study design	Sample size	Age	M	Sample size	Age	M	Aortic valve area (cm^2^)	Outcomes
Hase *et al*., 2021^14^	Japan	Observational	69	86 ± 5.3	50	138	86.3±3.8	15	361.1 (332.5–388.0)	Mortality Stroke Bleeding PPM AKI
Wahab *et al*., 2020^7^	Germany	RCT	121	81.9 ± 6.7	52	120	79.6 (15.8)	34	0.7 (0.2)	(5 years) Mortality Stroke Hospitalization MI Bleeding PPM
Wahab *et al.*, 2014^9^	Germany	RCT	121	81.9 ± 6.7	52	117	79.6 (15.8)	34	0.7 (0.2)	(30 days) Mortality Stroke Hospitalization MI Bleeding PPM AKI
Wahab *et al*., 2015^8^	Germany	RCT	121	81.9 ± 6.7	52	117	79.6 (15.8)	34	0.7 (0.2)	(1 year) Mortality Stroke Hospitalization MI Bleeding PPM
Mauri *et al*., 2017^15^	Germany	Multicenter propensity-matched	92	82.8 ± 6.3	11	92	81.8±5.0	9	<400 mm^2^	30-day mortality 1-year mortality Stroke Bleeding PPM
Deharo *et al*., 2020^16^	France	Propensity score matching	10459	83.04 ± 6.57	10459	10459	83.09±6.43	4240	N/A	Mortality Stroke Hospitalization
Lanz *et al*., 2019^10^	Switzerland, UK, Germany	RCT	364	83·0 ± 3·9	165	374	82·6 (4·3)	154	0·7 (0·2)	(30 days) Mortality Stroke Bleeding AKI MI PPM
Kalogeras *et al*., 2023^17^	UK	Retrospective	756	80.9 ± 7.5	435	917	81.5±7.1	432	78 (73–84)	PPM AKI Bleeding
Mosleh *et al*., 2023^18^	UK	Retrospective	337	83.1 ± 7.5	26	236	82.7±7.9	20	0.7±0.2	Mortality Stroke PPM Bleeding
Rhuede *et al*., 2022^20^	Germany	multicenter, propensity-matched comparison	467	82 [77–85]	229	467	82 [78–85]	225	BEV= 75 [70–80] SEV= 75 [71–78]	Mortality PPM Stroke Bleeding MI AKI
Barki *et al*., 2022^21^	Italy	Retrospective cohort analysis	58	82 ± 6	29	108	83 ± 5.7	66	BEV= 0.7 ± 0.18 SEV= 0.75 ± 0.15	Mortality MI PPM Hospitalization Stroke Bleeding AKI
Costa *et al*., 2021^19^	Italy	Prospective cohort	548	82.3 ± 5.6	187	548	82.2 ± 5.9	181	BEV= 0.6 ± 0.2 SEV= 0.6 ± 0.3	Mortality Hospitalization MI Stroke
Fakhuda *et al*., 2021^22^	Japan	Retrospective cohort	5276	84.6 ± 5.1	1654	1550	85.1 [5.2]	409	N/A	Mortality Hospitalization PPM
Lee *et al*., 2021^23^	Korea	Retrospective cohort	25	81.7 ± 3.4	4	45	81.7±5.4	1	22.2±1.0	Mortality Stroke Bleeding AKI Stroke MI Hospitalization PPM
Kooistra *et al*., 2021^24^	Netherlands	Retrospective cohort	230	80.4 ± 7.1	108	70	80.8 ± 5.8	28		Mortality Stroke Bleeding PPM
Giannini *et al*., 2020	Italy	Prospective cohort	408	82.4 ± 6.2	0 (female only)	461	82.7± 6.4	0 (female only)	BEV= 404.8±65.7 SEV= 395.8±81.3	Mortality MI Stroke Bleeding AKI
Barth *et al*., 2019^26^	Germany	Retrospective cohort	211	81 ± 6	209	209	81 ± 5	145	BEV= 459±93 (mm^2^) SEV= 458±68 (mm^2^)	Mortality Bleeding MI Stroke PPM
Husser *et al*., 2017^27^	Germany	Prospective cohort	622	81 ± 6	122	311	81 ± 6	277	BEV= 4.5 ± 0.8 SEV= 4.4 ± 0.6	Mortality Stroke AKI Bleeding PPM Periop
Roger *et al*., 2017^28^	USA	Prospective cohort	183	81 ± 9	53.6%	74	82 ± 8	39.2%	BEV= 0.7 ± 0.17 SEV= 0.68 ± 0.13	Mortality AKI Stroke Bleeding PPM
Kiramijyan *et al*., 2016^29^	USA	Prospective cohort	104	82.9 ± 7.5	47	119	80.3 ± 9.7	77	0.38 ± 0.1	Mortality Stroke Bleeding AKI PPM
Trigo *et al*., 2016	Canada	Prospective cohort	40	81.2 ± 6.5	1	22	82.0 ± 5.9	12	369.9 ± 36.6 mm^2^	Stroke MI Bleeding PPM
Portratz *et al*., 2022^31^	Germany	Retrospective cohort	170	82.9 ± 6.7	71	170	82.5 ± 5.1	74	0.69 ± 0.17	AKI Bleeding Mortality Peacemaker
Thiele *et al*., 2020^11^	Germany	RCT	219	81.5 ± 5.7	109	219	81.7 ± 5.3	105	BEV= 0.8 (0.6–0.9) SEV= 0.7 (0.6–0.9)	Mortality Stroke PPM
Nieuwkerk *et al*., 2021^12^	Netherlands	RCT	2360	81.3 ± 7.1	94	3050	81.3 ± 6.9	1228	BEV= 0.67 ± 0.20 SEV= 0.64 ± 0.20	Mortality Stroke MI Bleeding PPM
Vlastra *et al*., 2019^13^	Netherlands, Italy Spain France	RCT	4096	81.5 ± 7.1	1732	4096	81.3 ± 7.1	1760	N/A	Mortality Stroke MI Bleeding PPM
Steinvil *et al*., 2018	Israel	Retrospective cohort	223	81(77–85)	190	512	83(79–87)	65	BEV= 23 (21–29) SEV= 23 (20–28)	Mortality AKI Bleeding Stroke PPM

AKI, acute kidney injury, BEV, balloon-expandable valve, M, male; MI, myocardial infarction; N/A, not available, PPM, permanent pacemaker, RCT, randomized controlled trial, SEV, self-expandable valve.

### Risk-of-bias assessment

According to the RoB2 tool, six of the seven RCTs explained the method of random sequence generation sufficiently^7–9,11–13^. Hence, they were considered a low risk of bias. One study was marked unclear due to a lack of sufficient data^10^. Random allocation concealment was reported in detail in five studies, and the risk of bias was marked as low in these studies^7–9,11,13^. Two studies showed some concerns and hence were marked as having a high risk of bias^10,12^. Most of our included studies reported the blinding methods used for the participants, personnel, or outcome assessment, so they were considered to have a low risk of bias. No study has any missing data. Regarding other sources of bias, all studies were considered as having a low risk of bias.

The quality assessment for observational studies, carried out by NOS, showed that none of the studies scored 8.

The quality assessment of the 7 RCTs and 19 non-randomized prospective studies is presented in Table 2.

**Table 2:** Risk of Bias for RCTs

Author (year)	Random sequence generation	Allocation concealment	Selective reporting	Blinding of participants/personnel	Blinding of outcome assessment	Incomplete outcome data	Other sources of bias
Wahab *et al*., 2020^7^	Low risk	Low risk	Low risk	High risk	Low risk	Low risk	Low risk
Wahab *et al*., 2014^9^	Low risk	Low risk	Low risk	High risk	High risk	Low risk	Low risk
Wahab *et a*l., 2015^8^	Low risk	Low risk	Unclear	Low risk	Unclear	Low risk	Low risk
Lanz *et al*., 2019^10^	Unclear	High risk	Low risk	High risk	Unclear	Low risk	Low risk
Thiele *et al*., 2020^11^	Low risk	Low risk	Low risk	Low risk	Low risk	Low risk	Low risk
Nieuwkerk *et al*., 2021^12^	Low risk	High risk	High risk	High risk	High risk	Low risk	Low risk
Vlastra *et al*., 2019^13^	Low risk	Low risk	Unclear	Low risk	Unclear	Low risk	Low risk

RCT, randomized controlled trial.

### Publication bias

**Figure 2:**
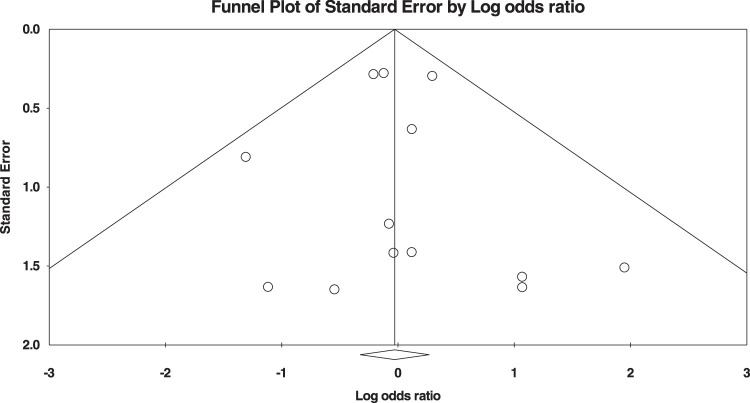
Funnel plot.

The publication bias between studies is illustrated in Figure 2.

### Quantitative analysis

The final 23 articles were analyzed for our desired outcomes. Some studies reported our desired outcomes more than once.

#### Primary outcomes

**Figure 3:**
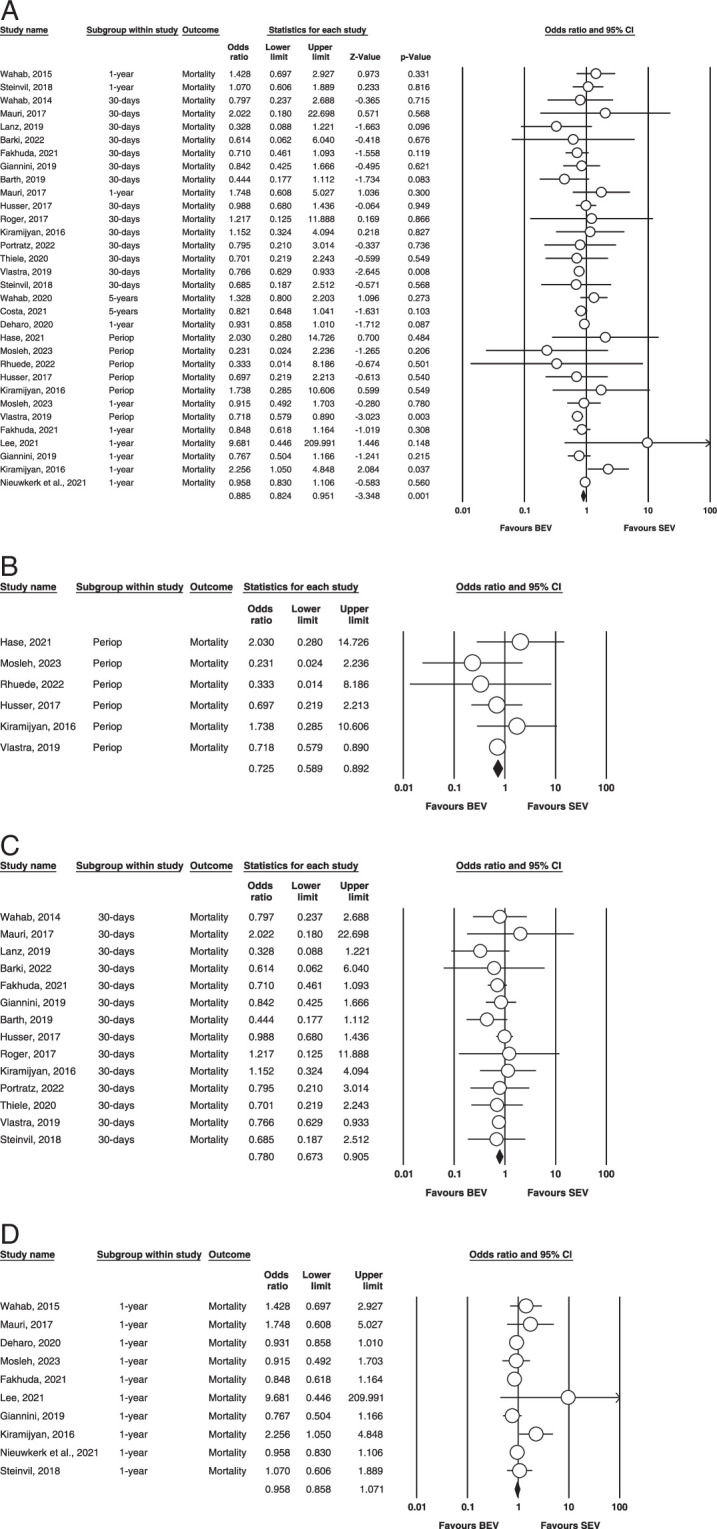
(A) Overall mortality. (B) Perioperative mortality. (C) 30-days mortality. (D) 1-year mortality. BEV, balloon-expandable valve; SEV, self-expanding valve.

Mortality (Figure 3A–D) illustrates the outcome.

Overall: A total of twenty-one studies^7–16,18,19,21–23,25–29,31,32^ were included that assessed mortality. Our analysis found that the results were significant for BEV compared to SEV for overall mortality. (OR=0.885, 95% CI= 0.824–0.951 *P*=0.001). It was found to be mild to calculate heterogeneity via fixed effect mode (*I^2^* =14.5% *P*=0.273).

Perioperative: A total of six^13,14,18,20,27,29^ out of twenty-six studies reported perioperative mortality. Our analysis found that the BEV group, when compared to SEV, was statistically significant (OR = 0.725; 95% CI: 0.589–0.892; *P* = 0.002) between the groups. No heterogeneity was found between studies in this analysis (*I^2^* = 0%; *P* = 0.677).

30 days: Fourteen studies^9–11,13–15,21,22,25–29,31^ reported 30-day mortality as one of their outcomes. A significant statistical relationship was found, and our analysis revealed favorability towards the BEV arm (OR = 0.780; 95% CI: 0.673–0.905; *P* = 0.001). No heterogeneity was found between our studies (*I^2^* =0, *P*=0.941) (Fig. 3).

1 year: A total of ten studies included this outcome^8,12,15,16,18,22,23,25,29,32^. There were no statistically significant differences in mortality between BEV and SEV. (OR = 0.958; 95% CI: 0.858–1.071; *P* = 0.448), and evidence of mild heterogeneity was found among the included studies (*I^2^* = 21.7%; *P* = 0.243) (Figure 3).

5 years: Only two studies reported and compared 5-year mortality between both groups; hence, no analysis was done.

**Figure 4:**
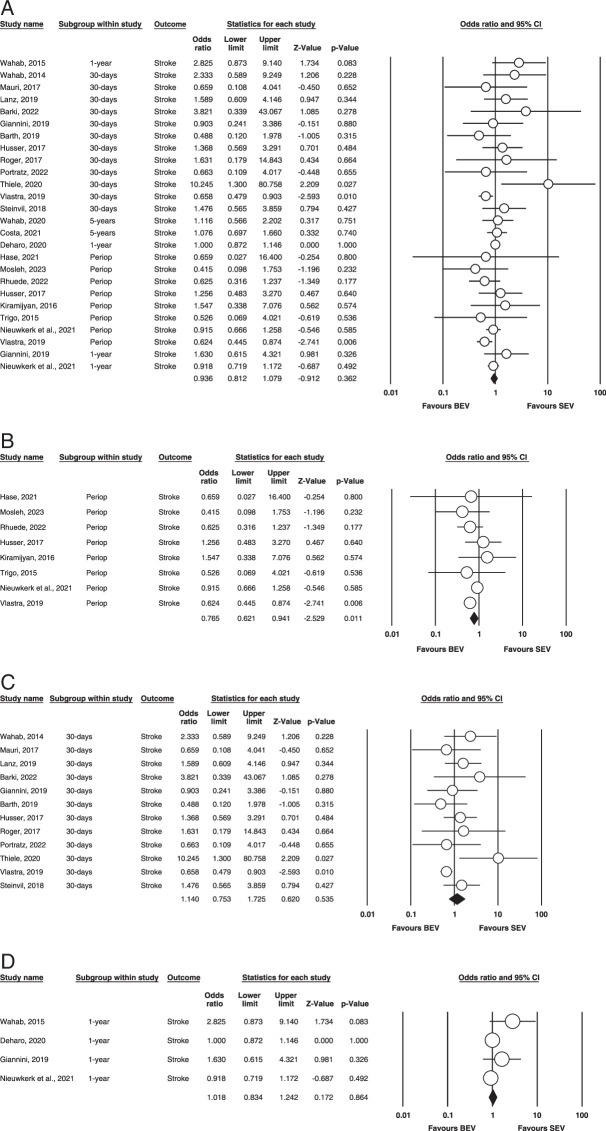
(A) Overall stroke. (B) Perioperative stroke. (C) 30-days stroke. (D) 1-year stroke. BEV, balloon-expandable valve; SEV, self-expanding valve.

Perioperative stroke. Figure 4 (A,B,C,D) illustrates the outcome.

Overall: A total of twenty-five studies were included that assessed stroke^7–16,18–21,25–32^. Our analysis found that the results were non-significant and none of the groups had more stroke incidence. (OR=0.936, 95% CI= 0.812–1.079 *P*=0.362). On calculating heterogeneity via fixed effect mode, it was found to be mild. (*I^2^* =21.3% *P*=0.162).

Perioperative: Eight studies reported this outcome^12–14,18,20,27,29,30^. Our analysis revealed no statistical significance (OR = 0.765; 95% CI: 0.621–0.941; *P* = 0.011) between the groups regarding stroke. Our analysis found no heterogeneity between studies (*I^2^* = 0; *P* = 0.582). The outcome is illustrated in (Figure 4).

30 days: In our meta-analysis, twelve studies evaluated this outcome^9–11,13,15,21,25–28,31,32^. There were no statistically significant differences in BEV vs. SEV for 30-day stroke (OR = 1.140; 95% CI: 0.753–1.725; *P* = 0.535), and small evidence of heterogeneity was found among the included studies (*I^2^* = 34%; *P* = 0.116) (Fig. 4).

1 year: Four of our included studies had this outcome^8,12,16,25^. There were no statistically significant differences between BEV and SEV for stroke after a year. (OR = 1.018; 95% CI: 0.834–1.242; *P* = 0.864), and evidence of heterogeneity was found among the included studies (*I^2^* = 32.4%; *P* = 0.218) (Figure 4).

5 years: Only two studies reported and compared 5-year stroke adverse events between both groups; hence, no analysis was done.

**Figure 5:**
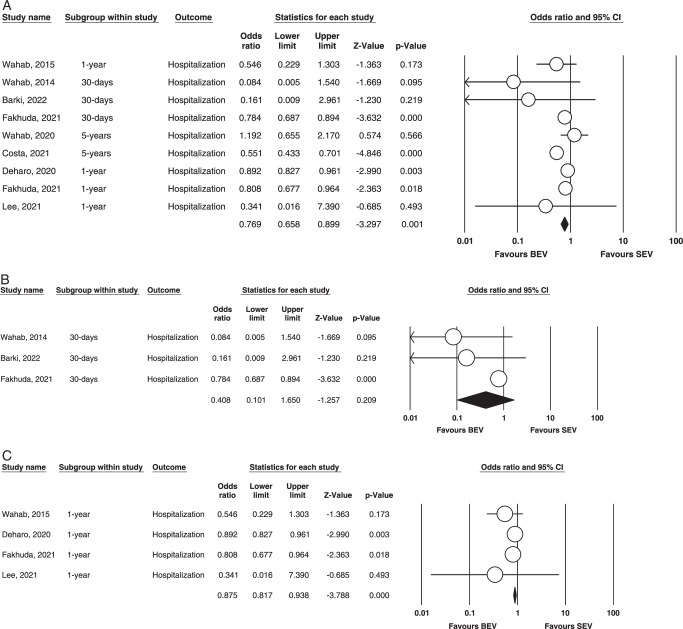
(A) Overall hospitalization. (B) 30-days hospitalization. (C) 1-year hospitalization. BEV, balloon-expandable valve; SEV, self-expanding valve.

Hospitalization. Figure 5 (A,B,C) illustrates the outcome.

Overall: A total of six studies were included that assessed hospitalization^7–9,16,19,21–23^. In our analysis, we found that the results were significant, favoring BEV. (OR=0.769 95% CI= 0.658–0.899 *P*=0.001). On calculating heterogeneity via fixed effect mode, it was found to be moderate. (*I^2^* =58.3% *P*=0.010).

30 days: Three studies reported this outcome^9,21,22^. Our analysis revealed that no statistical significance (OR = 0.408; 95% CI: 0.101–1.650; *P* = 0.209) was found between the groups when it came to hospitalization after BEV vs. SEV. We found moderate heterogeneity between studies during our analysis (*I^2^* = 41%; *P* = 0.184). The outcome is illustrated in Figure 5.

1 year: In our meta-analysis, four studies evaluated this outcome^8,16,22,23^. There were statistically significant differences in BEV vs. SEV for rates of hospitalization at one year, favoring BEV. (OR = 0.875; 95% CI: 0.817–0.938; *P* = 0.000), and no evidence of heterogeneity was found among the included studies (*I^2^* = 0; *P* = 0.474). (Fig. 5).

5 years: Only two studies reported and compared 5-year hospitalization between both groups; hence, no analysis could be performed.

#### Secondary outcomes

**Figure 6:**
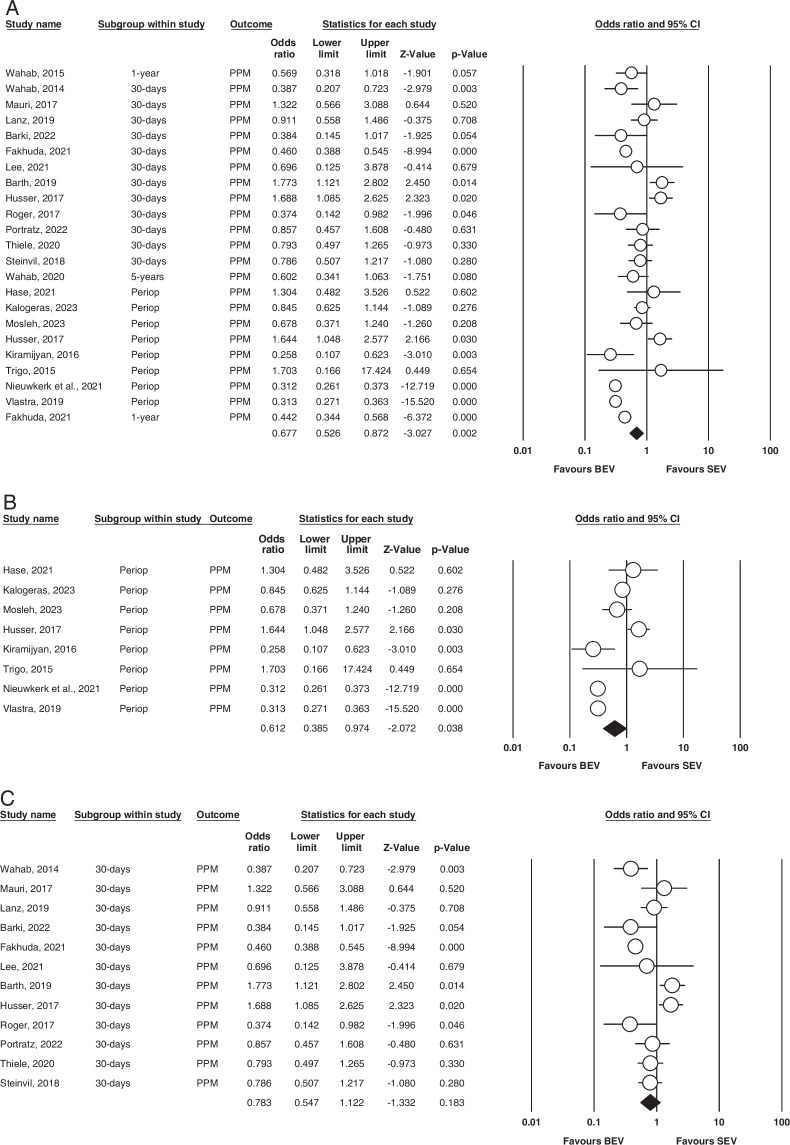
(A) Overall PPM. (B) Perioperative PPM. (C) 30-days PPM. BEV, balloon-expandable valve; PPM, permanent pacemaker; SEV, self-expanding valve.

Permanent pacemaker (PPM). Figure 6 (A, B, C) illustrates this outcome.

Overall: A total of nineteen studies were included that assessed PPM^7–15,17,18,21–23,26–31^. In our analysis, we found that the results were significant for BEV (OR=0.772, 95% CI= 0.526–0.872 *P*=0.002). It was found to be high in calculating heterogeneity via fixed effect mode (*I^2^* =88.2% *P*=0.000).

Perioperative: Eight studies reported this outcome^12–14,17,18,27,29,30^. Our analysis revealed slight statistical significance (OR = 0.612; 95% CI: 0.385–0.974; *P* = 0.038) between the groups when it came to perioperatively PPM implantation. BEV group was found to have been favored. Our analysis found no heterogeneity between studies (*I^2^* = 0; *P* = 0.000). The outcome is illustrated in Figure 6.

30 days: In our meta-analysis, twelve studies evaluated this outcome^9–11,15,21–23,26–28,31,32^. There were no statistically significant differences in BEV vs. SEV for 30-days PPM implantation (OR = 0.783; 95% CI: 0.547–1.122; *P* = 0.183), and strong evidence of heterogeneity was found among the included studies (*I^2^* = 82%; *P* = 0.000). (Fig. 4).

1 year and 5 years: Only two and one of our included studies had 1-year and 5-year outcomes, respectively; hence, no analysis was done.

**Figure 7:**
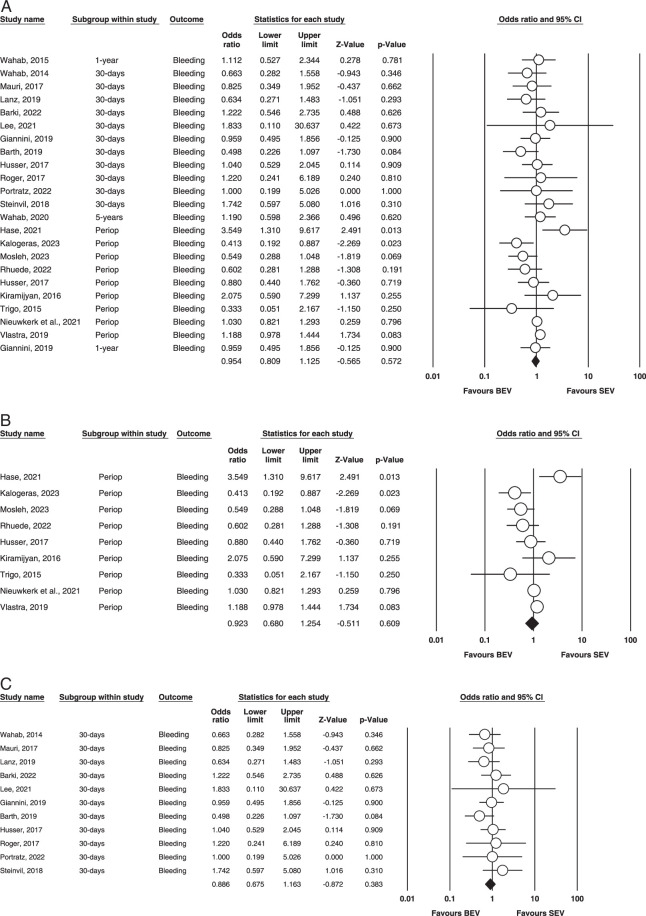
(A) Overall bleeding. (B) Perioperative bleeding. (C) 30-days bleeding. BEV, balloon-expandable valve; SEV, self-expanding valve.

Bleeding: Figure 7 (A, B, C) illustrates this outcome.

Overall: A total of nineteen studies were included that assessed bleeding incidence^7–10,12–15,17,18,20,21,23,25–28,30–32^. Our analysis found that the results were non-significant, and no difference in bleeding incidences was recorded between SEV and BEV. (OR=0.954, 95% CI= 0.809–1.125, *P*=0.000). It was found to be mild to calculate heterogeneity via fixed effect mode. (*I^2^* =24.5% *P*=0.140).

Perioperative: Nine out of twenty-six studies reported perioperative Bleeding^12–14,17,18,20,27,29,30^. Our analysis found that the BEV and SEV groups had no statistical significance for bleeding incidence (OR = 0.923; 95% CI: 0.680–1.254; *P* = 0.609) between the groups. Significant heterogeneity was found between studies in this analysis (*I^2^* = 63.2%; *P* = 0.005).

30 days: Eleven studies reported this outcome^9,10,15,21,23,25–28,31,32^. Our analysis revealed no statistical significance (OR = 0.886; 95% CI: 0.675–1.163; *P* = 0.383) was found between the groups regarding bleeding 30 days after BEV vs. SEV. Our analysis found no heterogeneity between studies (*I^2^* =0; *P* = 0.819). The outcome is illustrated in Figure 5.

1 year and 5 years: Only two and one of our included studies had 1-year and 5-year outcomes, respectively; hence, no analysis was done.

**Figure 8:**
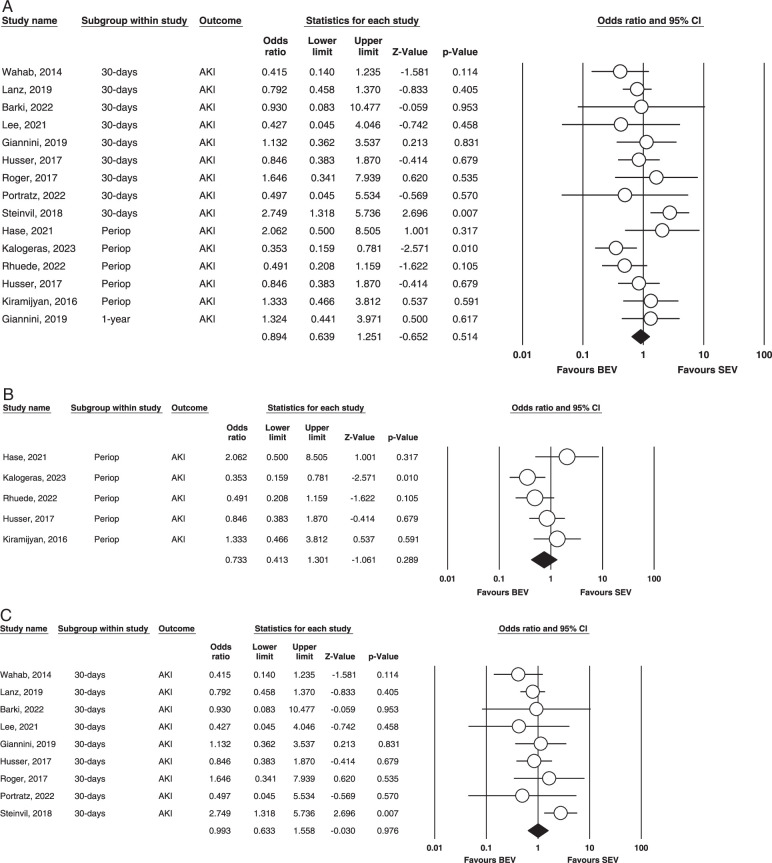
(A) Overall AKI. (B) Perioperative AKI. (C) 30-Days AKI. AKI, acute kidney injury; BEV, balloon-expandable valve; SEV, self-expanding valve.

Acute kidney injury (AKI). Figure 8 (A,B,C) illustrates this outcome.

Overall: Thirteen studies were found to assess AKI incidents^9,10,14,17,20,21,23,25,27–29,31,32^. Our analysis found that the results were non-significant (OR=0.894, 95% CI= 0.639–1.251, *P*=0.514). It was found to be mild to calculate heterogeneity via fixed effect mode. (*I^2^* =36.327% *P*=0.079).

Perioperative: Five studies reported this outcome^14,17,20,27,29^. Our analysis revealed no statistical significance (OR = 0.733; 95% CI: 0.413–1.301; *P* = 0.289) between the groups regarding the incidence of AKI. Our analysis found moderate heterogeneity between studies (*I^2^* = 45.9%; *P* = 0.116). The outcome is illustrated in Figure 3.5.

30 days: In our meta-analysis, nine studies evaluated this outcome^9,10,21,23,25,27,28,31,32^. There were no statistically significant differences in BEV vs. SEV for 30-days AKI incidence (OR = 0.993; 95% CI: 0.633–1.558; *P* = 0.976), and small evidence of heterogeneity was found among the included studies (*I^2^* = 32.9%; *P* = 0.154). (Fig. 4).

1 year and 5 years: Only one study had evaluated one year of AKI incidence, warranting no quantitative meta on it.

**Figure 9:**
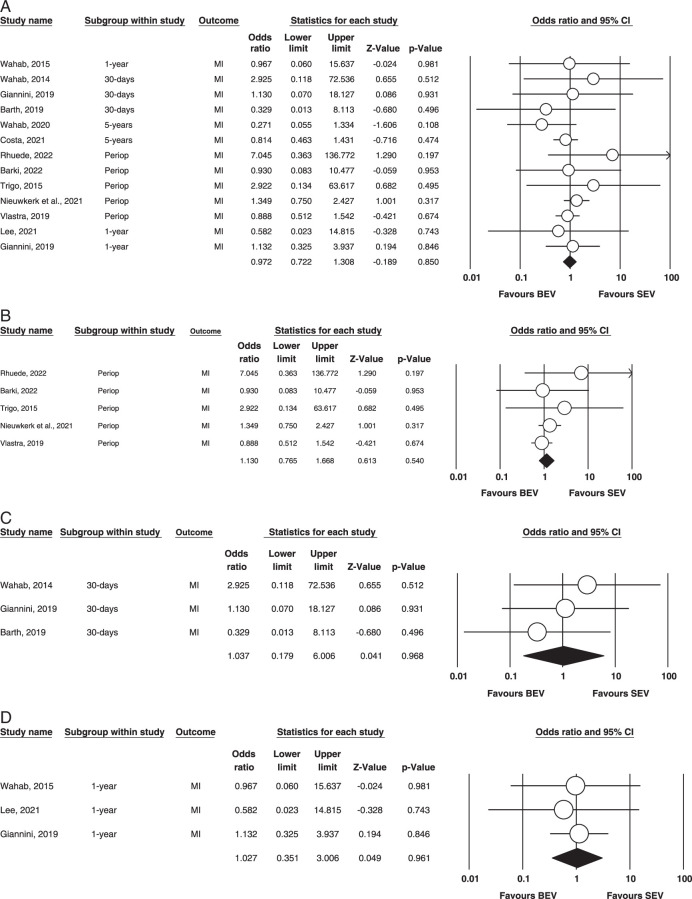
(A) Overall MI. (B) Perioperative MI. (C) 30-days MI. (D) 1-year MI. BEV, balloon-expandable valve; MI, myocardial infarction; SEV, self-expanding valve.

Myocardial infarction (MI). Figure 9 (A,B,C,D) illustrates this outcome.

Overall: Ten studies evaluated Mi, post TAVR surgeries^7–9,12,13,19–21,23,25,26,30^. Our analysis found that the results were non-significant (OR=0.972, 95% CI= 0.722–1.308, *P*=0.850). No heterogeneity was calculated via fixed effect mode. (*I^2^* =0 *P*=0.829).

Perioperative: Five studies reported this outcome^12,13,20,21,30^. Our analysis revealed no statistical significance (OR = 1.130; 95% CI: 0.765–1.668; *P* = 0.540) between the groups regarding MI. Our analysis found no heterogeneity between studies (*I^2^* = 0; *P* = 0.569). The outcome is illustrated in Figure 3.5.

30 days: In our meta-analysis, only three studies evaluated this outcome^9,25,26^. There were no statistically significant differences in BEV vs. SEV for the 30-day occurrence of MI (OR = 1.037; 95% CI: 0.179–6.006; *P* = 0.535), and no evidence of heterogeneity was found among the included studies (*I^2^* = 0; *P* = 0.968). (Fig. 4).

1 year: Three of our included studies had this outcome^8,23,25^. There were no statistically significant differences between BEV and SEV for stroke after a year. (OR = 1.027; 95% CI: 0.351–3.006; *P* = 0.961), and no evidence of heterogeneity was found among the included studies (*I^2^* = 0; *P* = 0.931). (Fig. 3)

5 years: Only two studies reported and compared 5-year MI between both groups; hence, no analysis was done.

## Discussion

During the last few decades, minimal techniques have taken the world by storm due to numerous advantages with minimal blood loss, fewer complications, and shorter hospital stays, among many others^33^. In the context of Aortic stenosis, transcatheter is being compared and studied against surgical valve replacement^3,34^. Multiple meta-analyses have evaluated the BEV and SEV for different outcomes in transcatheter aortic replacement. However, we found that most studies evaluated either short-term outcomes, procedural success, or aortic function restoration, while one study compared long-term and short-term outcomes^34–38^. Our paper is the first to consider combining and subgrouping outcomes at a particular time frame for analysis.

Our analysis also focused on outcomes between BEV and SEV without restriction for the type of study and stratification only based on outcome. Hence, it may be an analysis with the most significant number of studies. Our first outcome of interest mortality was significant and favored BEV over SEV for at least three subgroups. This was contrary to previous literature that found none of the valve systems superior to another^35,37^. However, a recently published analysis favored BEV regarding short-term overall mortality^38^. Notable is the point that while our study had no statistical significance for a year’s mortality, the overall mortality favored BEV, making it a better choice. For our next outcome, stroke, none of the groups were favored overall or any of the subgroup analyses. This was in line with previous investigations and results^36,37^. This could lead to the hypothesis that if stroke is not the leading cause of mortality after TAVR in any group, other non-apparent causes need investigation. Our subsequent leading outcome of interest, hospitalization, was not a part of many previous investigations. However, we found it statistically significant as an overall outcome and favoring BEV. This could point out that fewer complications were associated with BEV in general. Even if there were more, the recovery time was faster, benefiting the patients undergoing this procedure overall.

For PPM, our outcomes and their results were in line with previously reported literature. Our analysis favored BEV for having fewer incidents of pacemaker implantation compared to SEV^37,38^. However, none of the groups were superior for the rest of our outcomes (Bleeding Risk, AKI, and MI). This can also explain that the mortality difference seen and found in our analysis and previous literature may be a complex attribute and may be related to procedural failure, iatrogenic or individual patient comorbidities. However, this leaves room for future research to determine the ultimate leading causes of mortality when general complications that follow surgery are non-significant.

Despite the considerable attention surrounding TAVR and its subtypes, only a few of our examined outcomes exhibited statistical significance. Hence, it sheds light on the fact that choosing a specific valve type needs more than just looking into its features. The healthcare providers may want to investigate availability, affordability, technical considerations, and factors influencing postoperative management to optimize outcomes, make informed decisions, and minimize the need for additional interventions.

Our paper has some limitations. The first limitation is the involvement of specific companies in manufacturing each type of valve, introducing potential sources of conflict. Secondly, one of our included studies, Deharo *et al*.^16^, had a large sample size compared to other groups, influencing results. The study focused on specific outcomes, including mortality, stroke, hospitalization, MI, bleeding, AKI, and pacemaker insertion to maintain clinical relevance and statistical power. This approach was chosen due to resource constraints and the need to manage heterogeneity among included studies. Consequently, other essential factors, such as vascular complications, infections, and surgical success, were not analyzed. While this streamlined focus enhances the applicability of the findings to clinical practice and aligns with existing literature, it is essential to recognize that a more comprehensive assessment of all possible outcomes would offer a broader perspective on TAVR outcomes. Additionally, most of our included studies were observational. While observational studies provide valuable real-world data, they inherently carry limitations related to potential biases and confounding variables. The absence of RCTs may limit the ability to establish causal relationships between valve type (self-expanding or balloon-expandable) and the selected outcomes. Therefore, while our findings offer valuable insights, they should be interpreted in the context of the inherent limitations of observational study designs.

## Conclusion

In conclusion, our analysis accurately evaluates the outcomes of BEV vs. SEV. While our findings suggest potential advantages of BEV in terms of mortality and fewer hospitalizations, further research is still warranted to evaluate the two options. Particularly considering the non-significant results for other outcomes, cost-effectiveness, and patient-reported outcomes to inform clinical decision-making and enhance patient care in this surgical domain.

## Ethics statement

No ethical approval was required for this Systematic review.

## Consent

Informed consent was not required for this systematic review.

## Source of funding

No financial support was received for the conduct of this study.

## Author contribution

Q.A.K., A.M.F., and A.B. conceptualize the study. M.A., N.F.B., and D.L. did the literature search and extracted the data. Q.A.K. and M.A. analyzed the data, and B.S. A.M.R.R., A.N., M.A., M.B. and A.M.F. wrote the original manuscript. A.B. and Q.A.K. critically revised and edited the final manuscript. All authors reviewed and approved the final manuscript before submission.

## Conflicts of interest disclosure

The authors declare that they have no conflicts of interest or financial interests related to the material of this manuscript.

## Research registration unique identifying number (UIN)

Registery used: PROSPERO Unique identifying number or registration ID: CRD42023456595 https://www.crd.york.ac.uk/prospero/record_email.php.

## Guarantor

Dr.Qaisar Ali Khan.

## Data availability statement

Data can be available upon reasonable request to the corresponding author.

## Provenance and peer review

Not commissioned, externally peer-reviewed.
